# SLC26A4 correlates with homologous recombination deficiency and patient prognosis in prostate cancer

**DOI:** 10.1186/s12967-022-03513-5

**Published:** 2022-07-14

**Authors:** Cong Luo, Zhi Liu, Yu Gan, Xiaomei Gao, Xiongbing Zu, Ye Zhang, Wenrui Ye, Yi Cai

**Affiliations:** 1 Department of Urology, Disorders of Prostate Cancer Multidisciplinary Team, Xiangya Hospital, Central South University, No. 87 Xiangya Road, Changsha, 410008 Hunan People’s Republic of China; 2 National Clinical Research Center for Geriatric Disorders, Xiangya Hospital, Central South University, No. 87 Xiangya Road, Changsha, 410008 Hunan People’s Republic of China; 3 Department of Urology, The Second Affiliated Hospital of Guizhou Medical University, Kaili City, 556000 Guizhou People’s Republic of China; 4 Department of Pathology, Disorders of Prostate Cancer Multidisciplinary Team, Xiangya Hospital, Central South University, No. 87 Xiangya Road, Changsha, 410008 Hunan People’s Republic of China; 5 Department of Oncology, NHC Key Laboratory of Cancer Proteomics, Xiangya Hospital, Central South University, Hunan Province, No. 87 Xiangya Road, Changsha, 410008 People’s Republic of China; 6 Department of Neurosurgery, Xiangya Hospital, Central South University, No. 87 Xiangya Road, Changsha, 410008 Hunan People’s Republic of China

**Keywords:** Homologous recombination deficiency, Prostate cancer, SLC26A4, Prognosis, PARP inhibitors

## Abstract

**Background:**

Homologous recombination deficiency (HRD) is closely associated with patient prognosis and treatment options in prostate cancer (PCa). However, there is a lack of quantitative indicators related to HRD to predict the prognosis of PCa accurately.

**Methods:**

We screened HRD-related genes based on the HRD scores and constructed an HRD cluster system to explore different clinicopathological, genomic, and immunogenomic patterns among the clusters. A risk signature, HRDscore, was established and evaluated by multivariate Cox regression analysis. We noticed that *SLC26A4*, a model gene, demonstrated unique potential to predict prognosis and HRD in PCa. Multi-omics analysis was conducted to explore its role in PCa, and the results were validated by qRT-PCR and immunohistochemistry.

**Results:**

Three HRD clusters were identified with significant differences in patient prognosis, clinicopathological characteristics, biological pathways, immune infiltration characteristics, and regulation of immunomodulators. Further analyses revealed that the constructed HRDscore system was an independent prognostic factor of PCa patients with good stability. Finally, we identified a single gene, *SLC26A4*, which significantly correlated with prognosis in three independent cohorts. Importantly, *SLC26A4* was confirmed to distinguish PCa (AUC for mRNA 0.845; AUC for immunohistochemistry score 0.769) and HRD (AUC for mRNA 0.911; AUC for immunohistochemistry score 0.689) at both RNA and protein levels in our cohort.

**Conclusion:**

This study introduces HRDscore to quantify the HRD pattern of individual PCa patients. Meanwhile, *SLC26A4* is a novel biomarker and can reasonably predict the prognosis and HRD in PCa.

**Supplementary Information:**

The online version contains supplementary material available at 10.1186/s12967-022-03513-5.

## Introduction

Prostate cancer (PCa), a common malignant tumor, is the second leading cause of cancer-related mortality in men worldwide [1]. It can metastasize to bone (80%-100%), lymph nodes, liver, adrenal gland, or lung [2]. Although most early localized prostate cancer can achieve satisfactory results by prostatectomy or radiotherapy with a 5-year survival rate of 98.9%, metastasis is mainly found on initial diagnosis, hampering the avenue to a good prognosis. Therapy for metastatic PCa remains limited, and the current standard therapy is androgen deprivation therapy (ADT) combined with chemotherapy [3, 4]. Although ADT is initially effective, most patients inevitably develop into lethal metastatic castration-resistant prostate cancer (mCRPC) within 2–3 years [5], and the 5-year survival rate of them is only 28.2% [1]. Accordingly, there is significant enthusiasm to improve the stratification of patients with prostate cancer so that high-risk patients can be found earlier and receive active treatment.

The homologous recombination pathway plays a vital role in DNA repair and involves many genes [6], including *BRCA* (*BRCA1/2*), *ATM*, *CHEK2*, etc. Accumulated evidence has revealed the value of homologous recombination deficiency (HRD) in PCa, representing a high risk of PCa carcinogenesis and aggressiveness. A quarter of patients with recurrent or advanced PCa carry germline or somatic mutations in HRD-related genes [7]. The most commonly altered HRD-related gene in prostate cancer is *BRCA2*, with a prevalence of 5–6% at the germline level in mCRPC patients [8, 9]. A previous study revealed that *BRCA2* mutation carriers have a 5.0 to 8.6-fold increased risk and a 15% absolute risk of developing PCa [10, 11]. Moreover, *BRCA2* mutation carriers have higher progression rates from local to systemic disease, higher Gleason scores, shorter metastasis-free survival, and lower overall survival rates when compared to non-carriers [12–14]. In general, HRD is closely associated with a worse prognosis in PCa.

By extracting the HRD scores and other information from The Cancer Genome Atlas prostate adenocarcinoma cohort (TCGA-PRAD), we established an HRD signature to distinguish between high-risk and low-risk PCa patients. Through in-depth analysis, we identified and validated the protective effect of Solute Carrier Family 26 Member 4 (*SLC26A4*) in PCa, which may guide the application of poly(ADP-ribose) polymerase (PARP) inhibitors in PCa complementary to the commonly HRD-related gene mutations.

## Material and methods

### Prostate cancer datasets and preprocessing

Three open datasets with prostate cancer samples, multi-omics data, and complete clinical information were retrieved from the Cancer Genome Atlas (TCGA), Memorial Sloan Kettering Cancer Center (MSKCC), and Gene-Expression Omnibus (GEO) databases on August 22, 2021, including TCGA-PRAD [15], MSKCC-PRAD [16], and GSE116918 [17] cohorts. Then fragments per kilobase of exon model per million mapped fragments (FPKM) values were transformed into transcripts per kilobase million (TPM) values and log-transformed. The HRD, including loss of heterozygosity (LOH), telomeric allelic imbalance (TAI), and large-scale state transitions (LST), as well as gene-level copy numbers, PARADIGM integrated pathways, immune subtypes, gene-level non-silent mutation, were downloaded from Pan-Cancer (PANCAN) cohort in UCSC Xena (https://xenabrowser.net/) [18, 19]. Patients in the TCGA-PRAD cohorts without specific HRD scores were excluded for further analysis.

### Profiling of HRD-related genes

The HRD scores and genome-wide DNA damage footprints were updated on June 13, 2017. Since then, patients in the TCGA-PRAD cohorts without specific HRD scores were excluded for further analysis. We quartered patients in the TCGA-PRAD cohort according to the HRD scores. Quarters 1 and 4 were defined as the bottom HRD group and top HRD group, respectively. Differential analysis was performed based on the transcriptomic data of the two groups using the “limma” R package. Genes with | log2(fold change) |> 0.5 and p value < 0.05 were selected for subsequent univariate Cox analysis, and those significantly correlating with patient progression-free interval (PFI or PFS) were defined as HRD-related genes. Their mutational and expressional profiles were investigated. We also calculated their Spearman’s correlations based on their mRNA expression levels and displayed it as an intra-correlation plot.

### Unsupervised clustering for HRD-related genes

Unsupervised clustering analysis was applied to identify distinct HRD patterns based on the expression of the above prognostic HRD-related genes and classify patients for further analysis. The consensus clustering algorithm determined the number of clusters and their stability. We used the ConsensuClusterPlus package to perform the above steps, and 1000 repetitions were conducted to guarantee the stability of classification [20].

The mRNA expression level of each HRD-related gene was depicted among the clusters. Principal Component Analysis (PCA) and Kaplan–Meier survival analysis were performed to assess the power of clustering. The distributions of clinicopathological characteristics, including age at diagnosis, Gleason score, primary outcome, biochemical recurrence (BCR), pathologic T stage, pathologic N stage, original zone of cancer, and immune subtype, were evaluated across the clusters.

### Pathway quantification at transcriptomic and proteomic levels

The PARADIGM algorithm integrates pathway, expression, and copy number data to infer activation of pathway features within a superimposed pathway (SuperPathway) network structure. The SuperPathway system comprises 1387 constituent pathways from three pathway databases, NCI-PID, BioCarta, and Reactome (last updated 05/2013), containing 19K pathway features, representing 7369 genes, 9354 complexes, 2092 families, 82 RNAs, 15 miRNAs, and 592 abstract processes. This dataset is ssGSEA scores for 1387 constituent pathways [19].

Reverse-phase protein array (RPPA) data from the PANCAN cohort were used to calculate the pathway activity score of 10 cancer-related pathways. RPPA is a high-throughput antibody-based technique with procedures like Western blots. Proteins are extracted from tumor tissue or cultured cells, denatured by SDS, printed on nitrocellulose-coated slides, followed by an antibody probe. The terms included Apoptosis, Cell Cycle, DNA Damage Response, Epithelial-Mesenchymal Transition (EMT), Hormone a, Hormone b, PI3K/AKT, RTK, and TSC/mTOR pathways. In brief, RBN RPPA data were median-centered and normalized by the standard deviation across all samples for each component to obtain the relative protein level. The pathway activity score is then the sum of the relative protein level of all positive regulatory elements minus that of negative regulatory components in a particular pathway [21].

### Estimation of tumor purity and fractions of immune cells

Estimation of stromal and immune components and tumor purity in tumor tissues using expression data was achieved by the “ESTIMATE” R package [22]. Subsequently, the population abundance (fraction) of tissue-infiltrating immune and stromal cell populations was estimated by three well-known algorithms, including MCP counter (10 cell types) [23], ImmuneCellAI (24 cell types) [24], and Cibersort (22 cell types) [25].

### Essential molecular characteristics of the tumor

We extracted vital molecular features of malignant tumors from an integrated and in-depth bioinformatics study [26], including proliferation, leukocyte fraction, B cell receptor (BCR) evenness, T cell receptor (TCR) evenness, Th1, Th2, and Th17 cells, aneuploidy score, intratumor heterogeneity (ITH), single nucleotide variant (SNV) neoantigens, insertion-and-deletion (indel) neoantigens, cancer-testis antigen (CTA) score, homologous recombination defects, and fraction of altered genome. The microsatellite instability (MSI) MANTIS score was downloaded from cBioPortol for Cancer Genomics (https://www.cbioportal.org/).

### Immunomodulator identification and analysis

A list of 78 immunomodulatory genes was obtained from a previous study that curated them from a literature review performed by immuno-oncology experts within the TCGA immune response working group [26]. Corresponding median mRNA expression levels were used to summarize expression in each cluster. We performed a limma differential analysis across clusters to examine differences in immunomodulatory gene expression and found genes to be significantly differentially expressed. And the immunomodulatory copy number was also outputted from a PANCAN cohort as deep amplifications (2), shallow amplifications (1), non-alterations (0), shallow deletions (− 1), and deep deletions (− 2) of each immunomodulator gene. Proportions of samples with each type of copy number alteration were then compared across HRD clusters.

### Profiling of prognostic hub genes and dimensionality reduction

We performed differential expression analysis between pairs in this cohort of HRD clusters and performed Cox survival analysis after taking the intersection of the resulting differentially expressed genes. Those with survival significance were set as prognostic hub genes, whose expression patterns were employed as the basis of subsequent PCA analysis. The risk signature was termed as ‘HRDscore’ and calculated by the following formula:

$$HRDscore=\sum \left[\left({PC}_{1}+{PC}_{2}\right)\times {expression}_{risk}-\left({PC}_{1}+{PC}_{2}\right)\times {expression}_{protective}\right]$$

where “${expression}_{risk}$” stood for expression levels of risk genes and “${expression}_{protective}$” stood for that of protective genes.

Patients were dichotomized into high HRDscore, and low HRDscore groups based on the best cut-off decided by X-tile software. A Sankey plot was established to investigate the intrinsic relationship among HRD cluster, immune subtype, and HRDscore. Furthermore, we explored the correlations between the HRDscore and clinicopathological features, including survival. For subgroup analysis, TCGA-PRAD patients were divided into different groups based on features as follows: age (≤ 45 years old or > 45 years old) and Gleason score (< 8 or ≥ 8). Finally, multivariate Cox analyses were conducted to test the robustness of the established HRDscore.

### Prediction of immunotherapy response and correlation with immune cells

ImmuCellAI was used to predict the response of immune checkpoint blockade (ICB) therapy based on the transcriptomic data [24]. A receiver operating characteristic (ROC) curve was built to illustrate the power of HRDscore in predicting immunotherapy response.

We calculated the correlations between HRDscore and fractions of immune cells and the prognostic value of these cell types. Next, several genes were obtained after the intersection between HRD-related and prognostic hub genes. Their relationship to immune cells was also measured to find critical genes that bridge HRD scores, immune infiltration, and patient prognosis.

### Quantitative real-time PCR assay

Quantitative real-time PCR was performed with SYBR Green PCR mixture (Using Roche lightcycler 480 system) according to standard protocols. PCR conditions were: one cycle of 5 min at 95 °C, then 45 cycles of 10 s at 95 °C, 10 s at 60 °C, 10 s at 72 °C. The expression of the *SLC26A4* gene was normalized to the expression of the GAPDH gene using the comparative CT method. Primers used were: *SLC26A4* (F: 5′-AGGAAATATGCACTGCTCACT- 3′; R: 5′-AGTATTCCCGCAGTTTGCTGA-3′); *GAPDH* (F: 5′-CAAGGCTGAGAACGGGAAG-3′; R: 5′-TGAAGACGCCAGTGGACTC-3′).

### Prostate cancer samples and immunohistochemistry

Prostate cancer samples were acquired from Xiangya Hospital of Central South University. A physician obtained informed consent from the patients. The procedures related to human subjects were approved by the Ethics Committee of Xiangya Hospital, Central South University. Tissues were fixed in 10% buffered Formalin, then transferred to 70% alcohol. These paraffin-embedded tissues were sectioned (4 μm) and stained with antibodies against SLC26A4 (HPA042860, Atlas Antibodies). The following detection and visualization procedures were performed according to the manufacturer's protocol. To quantify the immunohistochemistry (IHC) result of positive staining, five random areas in each tissue sample were microscopically examined and analyzed by an experienced pathologist. The average staining score was calculated by dividing the positive areas by entire regions.

### Statistical analyses

The univariate and multivariate Cox analyses were performed to detect the prognostic factors. Kaplan–Meier curves with the log-rank test were used to assess survival differences between groups. Spearman correlation analyses were used to calculate correlations. The cutoff value was determined using the X-tile software (version 3.6.1). All statistical analyses were conducted using R software (version 4.1.2), and most visualization was achieved using the “ggplot2” R package. P < 0.05 was considered statistically significant.

## Results

### The landscape of genetic variation of HRD-related genes in prostate cancer

**Fig. 1:**
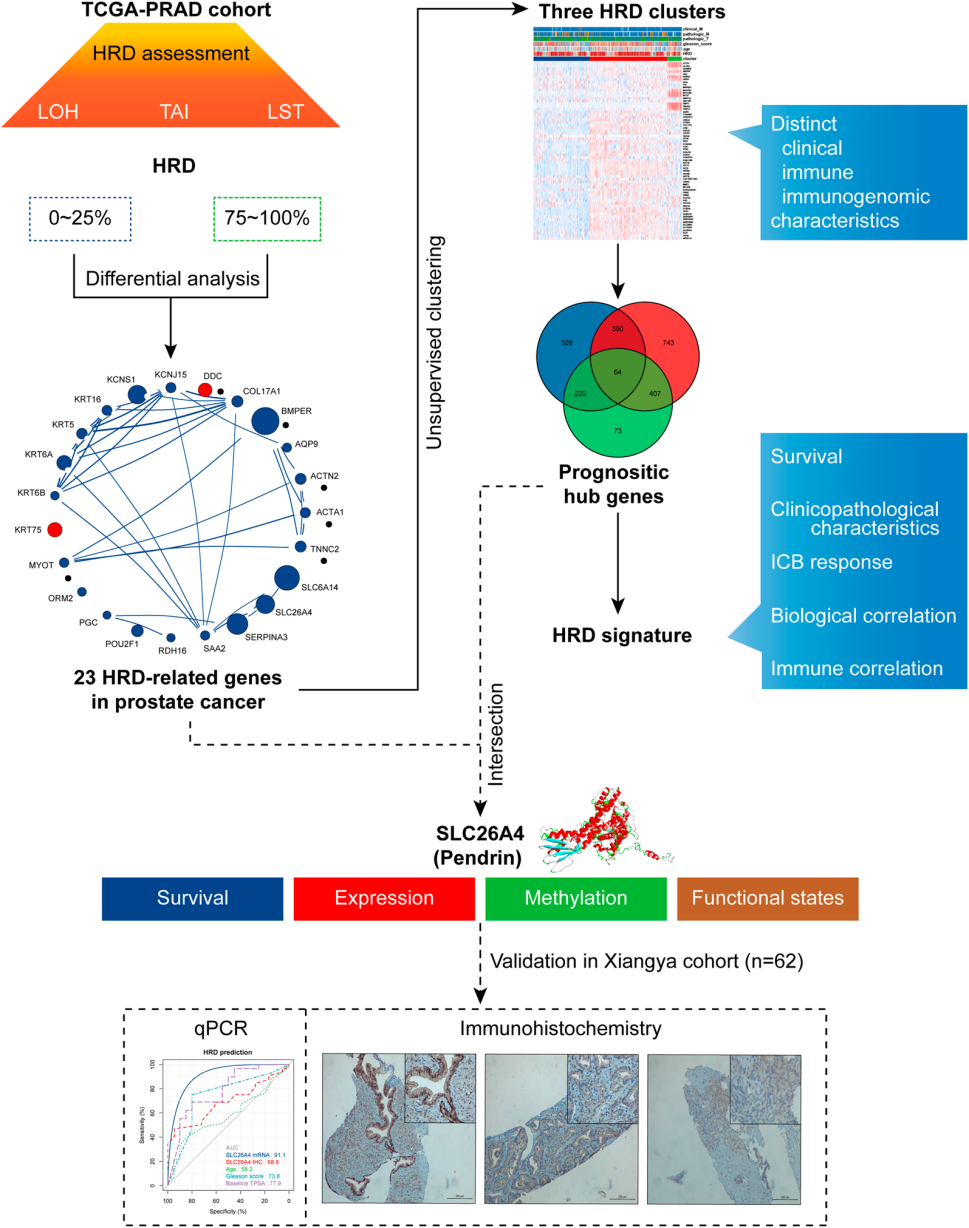
The flow chart of our analysis

**Fig. 2:**
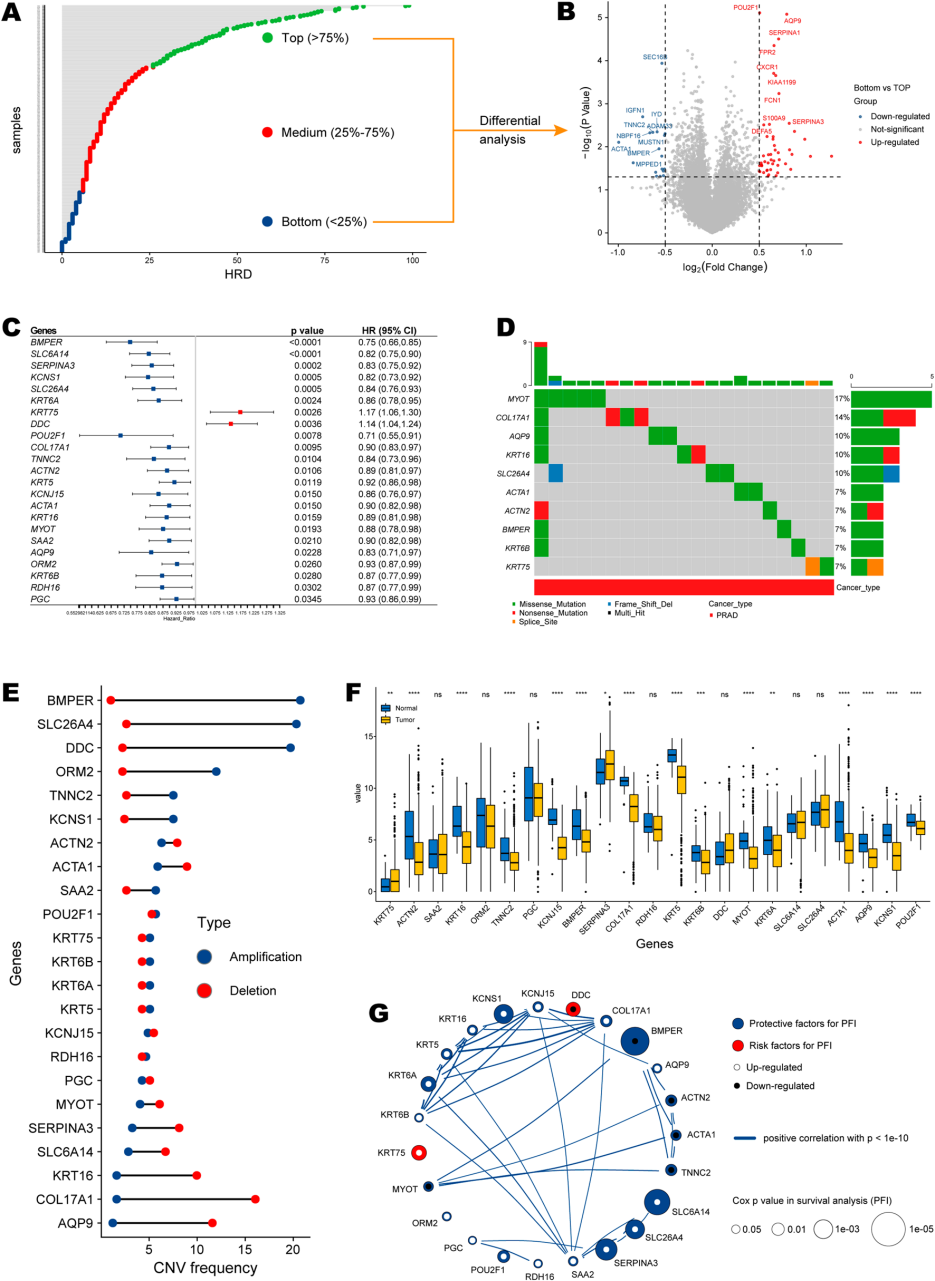
Profiling of HRD-related genes. **A** Distribution of prostate cancer samples according to HRD. Green dots meant the top HRD samples (Q1, > 75%), blue dots meant the bottom HRD samples (Q4, < 25%), red dots meant the medium HRD samples (Q2–3, 25% ~ 75%). **B** Volcano plot of differentially expressed genes between the top and bottom groups. **C** Forest plot of differentially expressed genes with significant prognostic value. Red block meant a risk effect (HR > 1), and blue block meant a protective effect (HR < 1). **D** Oncoplot showing the SNV mutation status of the TOP 10 HRD-related genes. **E** Dumbbell chart showing the CNV frequency of HRD-related genes. Red dots meant deletion frequency, and blue dots meant amplification frequency. **F** The expression differences of HRD-related genes between prostate cancer tissues and normal tissues. **G** The correlations among HRD-related genes. Red dots meant risk factors, and blue dots meant protective factors. The statistical significance is indicated as asterisks (*), *p < 0.05, ** p < 0.01, ***p < 0.001, ns: not significant

The flow chart of our study is summarized in Fig. 1. We first ranked the patients in the TCGA-PRAD cohort (n = 472) in order of HRD scores from high to low, and then subjected their transcriptomic data in the first and fourth quartiles (quartile 4 vs. quartile 1) to differential expression analysis (Additional file 4: Table S1; Fig. 2A, B). Sixty-six differential expressed genes were obtained, and subsequent univariate Cox analysis found 23 of them were prognostic (p < 0.05; Additional file 5: Table S2; Fig. 2C), defined as HRD-related genes.

Of the 23 HRD-related genes, most were protective factors for PFS of patients with prostate cancers, except KRT5 and DDC. Moreover, SLC26A4, KRT16, COL17A1, and AQP9 were genetically unstable. And briefly, the SNV frequencies in MYOT, COL17A1, AQP9, KRT16, and SLC26A4 were high, equal to or exceeding 10% (Fig. 2D). As for the copy number variation (CNV) status, BMPER, SLC26A4, and DDC shared a high amplification frequency, while KRT16, COL17A1, and AQP9 demonstrated a high deletion frequency (Additional file 6: Table S3; Fig. 2E). And most genes were differentially expressed between prostate cancer tissues and adjacent normal controls (Fig. 2F). The intra-correlation among these HRD-related genes was illustrated in Fig. 2G (Additional file 7: Table S4).

### Molecular patterns mediated by 23 HRD-related genes

**Fig. 3:**
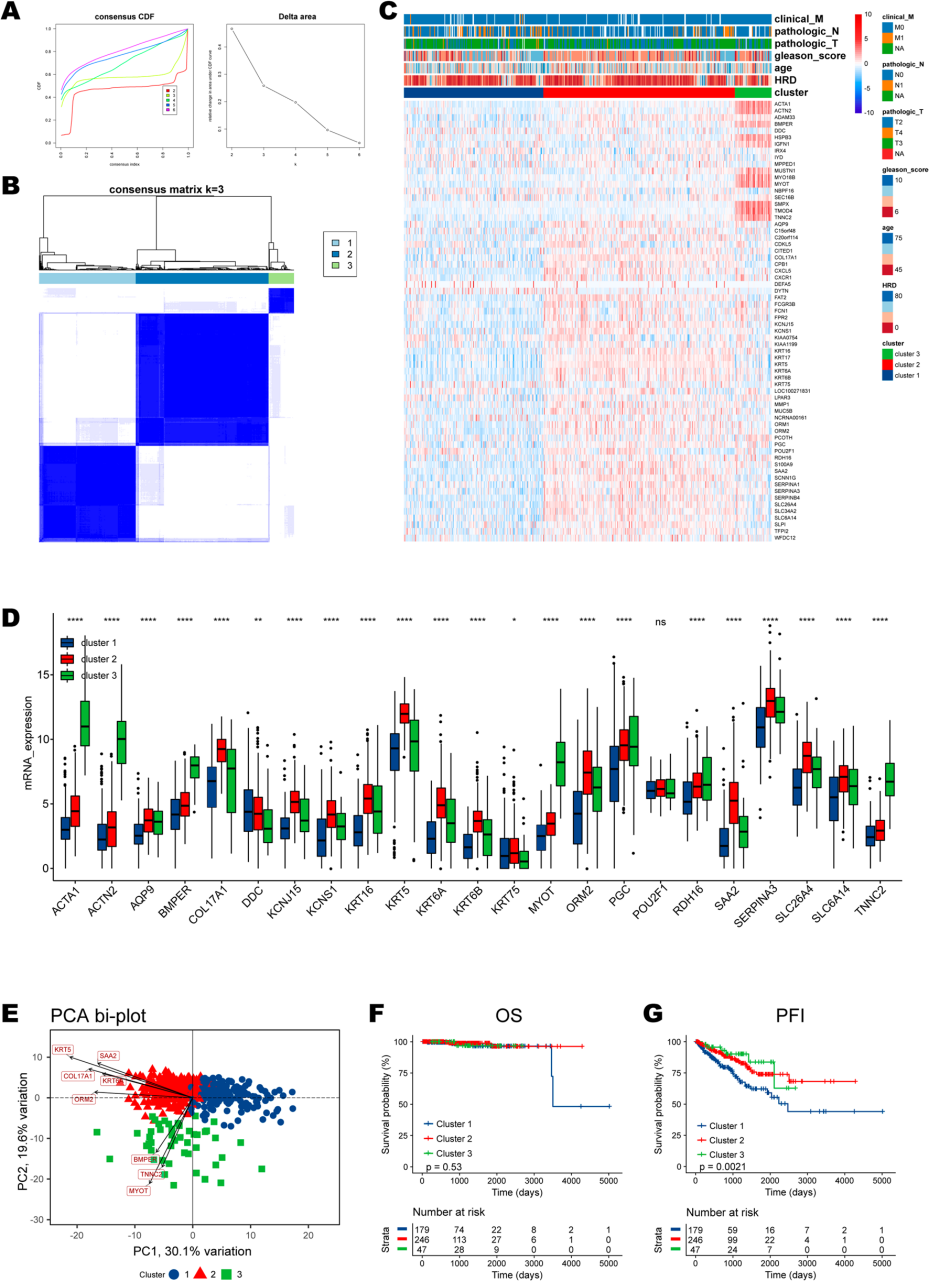
Consensus clustering of HRD-related genes. **A** CDF and delta area of consensus clustering. **B** The consensus matrix when k = 3. **C** Heatmap showing expression patterns of HRD-related genes and clinicopathological features in three clusters. **D** The detailed expression patterns of HRD-related genes in three clusters. **E** The PCA plot for the HRD cluster. **F**, **G** The survival differences in three clusters regarding OS and PFI. The statistical significance is indicated as asterisks (*), *p < 0.05, ** p < 0.01, ***p < 0.001, ns: not significant

An unsupervised clustering grouped patients from the TCGA-PRAD cohort into three distinct clusters based on the expression patterns of 23 HRD associated genes, including 179, 246, and 47 samples in the HRD clusters 1 to 3 respectively (Fig. 3A, B, E; Additional file 8: Table S5). The expression patterns of HRD-related genes and the distribution of clinicopathological features in the context of HRD clusters are displayed in Fig. 3C. We found that all genes were expressed at the lowest levels in HRD cluster 1 in both the heatmap and boxplot (Fig. 3C, D). Although the HRD cluster failed to distinguish patients’ overall survival (p = 0.53; Fig. 3F), cases in cluster 1 demonstrated the worst progression-free interval (p = 0.0021; Fig. 3G). Moreover, we clustered samples based on the expressions of 23 differentially expressed genes in the MSKCC-PRAD cohort and generated three clusters. Consistently, cluster 1 demonstrated the worst PFI (Additional file 1: Fig. S1).

### HRD clusters distinguish clinicopathological characteristics and biological pathways

**Fig. 4:**
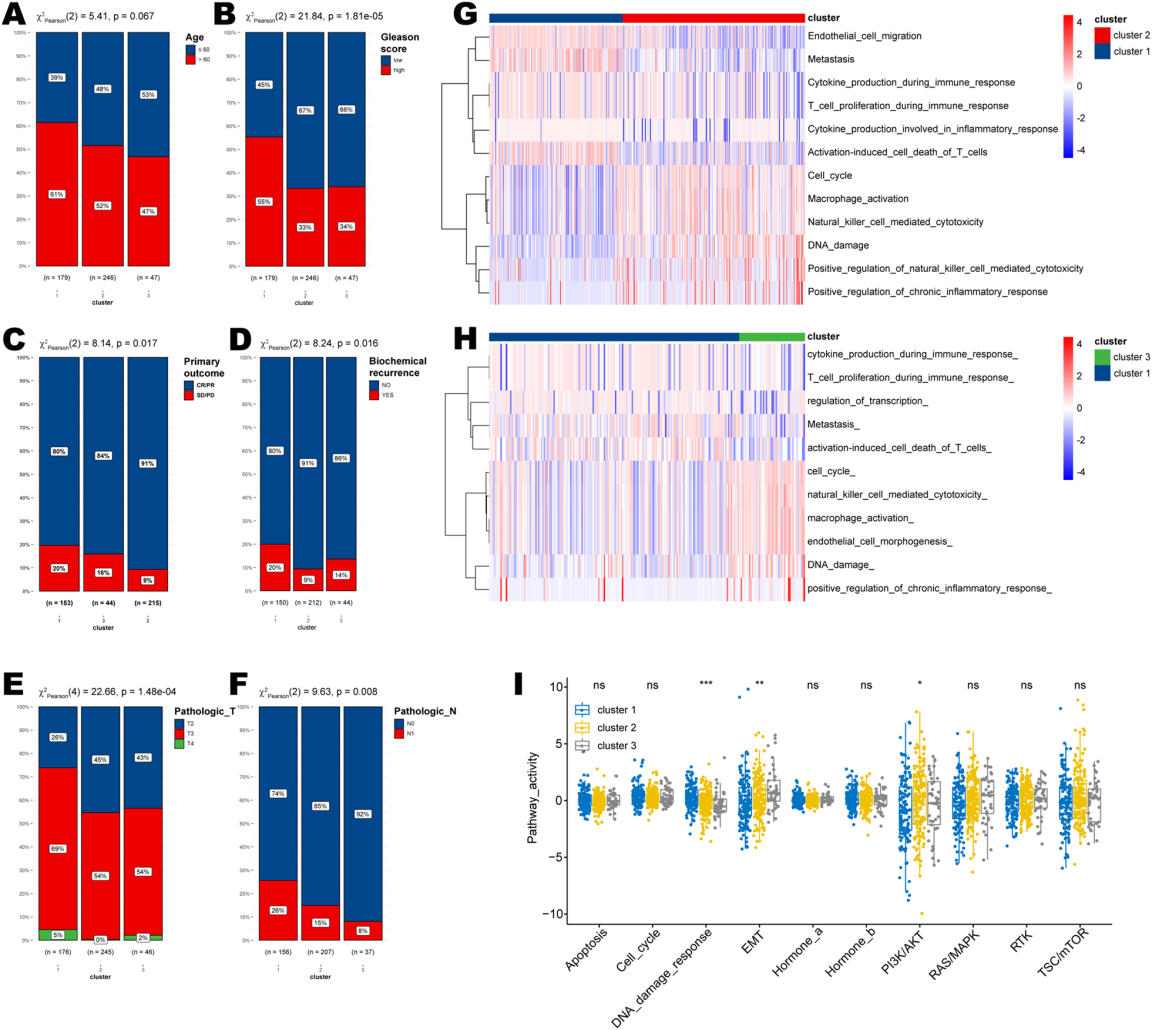
Clinicopathological features in HRD clusters. The differences in **A** patient age, **B** Gleason score, **C** primary outcome, **D** biochemical recurrence, **E** pathologic T stage, and **F** N stage in three HRD clusters. The differential PARADIGM pathways **G** between clusters 1 and 2, and **H** between clusters 1 and 3. **I** The differences in biological pathways at the protein level in HRD clusters. The statistical significance is indicated as asterisks (*), *p < 0.05, ** p < 0.01, ***p < 0.001, ns: not significant

Chi-square analyses among the three HRD clusters revealed that patients in cluster 1 harbored higher age and higher Gleason scores (Fig. 4A, B). Furthermore, prostate cancer samples in HRD cluster 1 showed more malignant properties as they displayed lower proportions of complete or partial response (CR/PR) but higher ratios of biochemical recurrence (Fig. 4C, D), as well as higher pathologic T and N stages (Fig. 4E, F).

Compared with clusters 2 and 3, HRD cluster 1 showed higher scores of pathways, including Metastasis, cytokine production during the immune response, T cell proliferation during the immune response, but lower scores of PARADIGM pathways such as cell cycle, macrophage activation, DNA damage, and natural killer cell-mediated cytotoxicity (Fig. 4G, H). Importantly, quantification of activated pathways using protein expression data also demonstrated that our HRD cluster could well reflect genetic alterations, as it uniquely distinguished patients regarding the pathway “DNA damage response” (p < 0.001; Fig. 4I).

### Immune infiltration characteristics in distinct HRD clusters

**Fig. 5:**
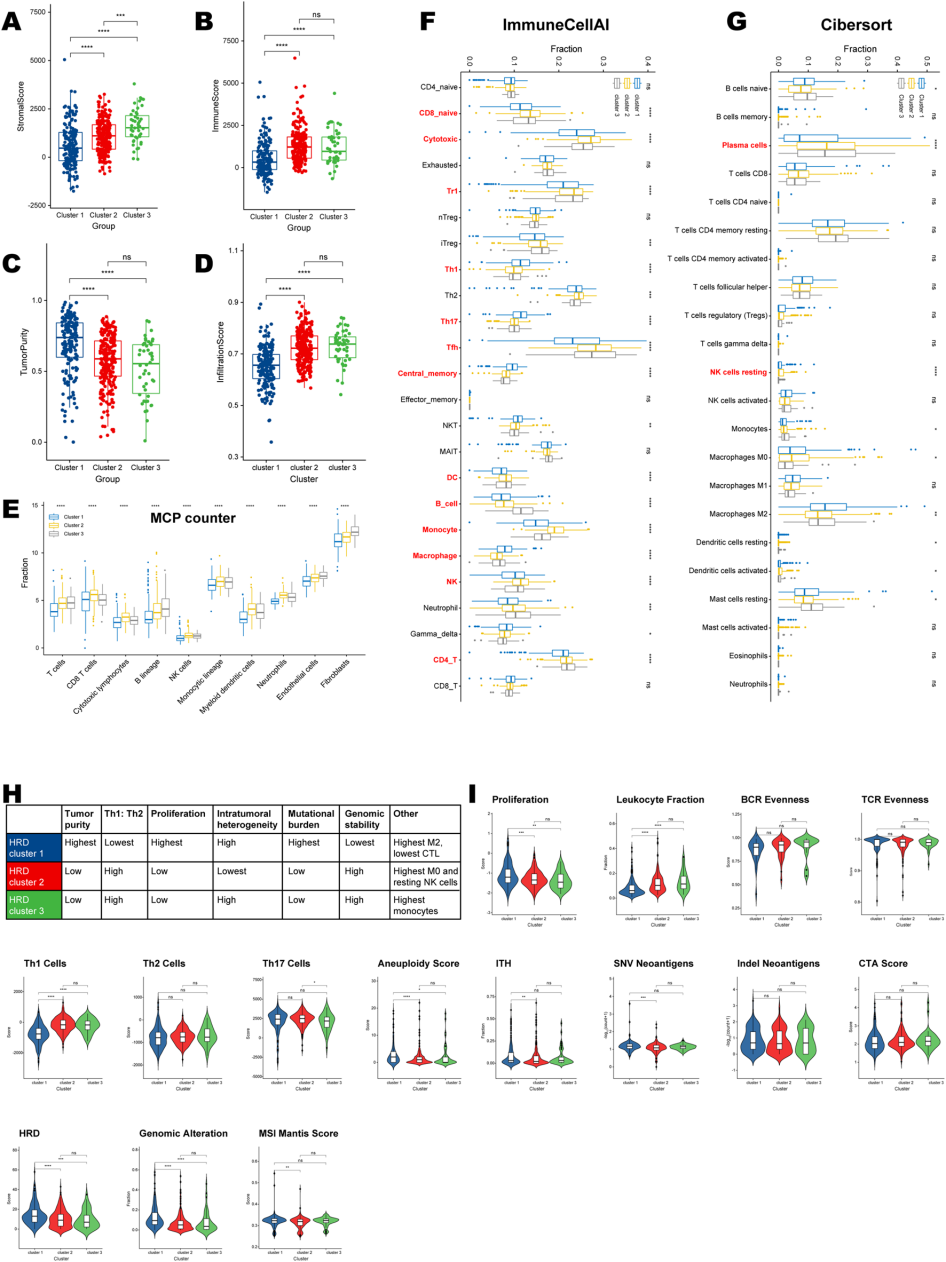
Immune and immunogenic features in HRD clusters. The differences in **A** stromal score, **B** immune score, **C** tumor purity, and **D** infiltration score in three HRD clusters. The immune infiltrating fractions in HRD clusters according to **E** MCP counter, **F** ImmuneCellAI, and **G** Cibersort algorithms. The cell types were set in red and bold if the corresponding p values for cluster differences were less than 0.0001 (****). The **H** summarized and **I** differences in HRD clusters' immunogenic features. Blue, red, and green meant HRD clusters 1–3, respectively

HRD cluster 1 distinctly showed the lowest stromal scores, immune scores, and highest tumor purity (Fig. 5A–C; Additional file 9: Table S6). Infiltration abundance estimated by ImmuneCellAI showed a consistent result, as cluster 1 had the lowest infiltration scores (Fig. 5D). Specifically, the fractions of every stromal or immune cell type were lowest in HRD cluster 1 (Fig. 5E–G; Additional file 9: Table S6). But there was a paradoxical situation where we observed that Th1, Th17, central memory cells, and macrophages were most abundant in cluster 1 (based on the ImmuneCellAI algorithm; Fig. 5F). Meanwhile, the fraction of M2 macrophage was highest in cluster 1 (based on the Cibersort algorithm; Fig. 5G).

The HRD clusters showed distinct genetic and immune signatures based on the dominant sample characteristics of their tumor samples (Fig. 5H, I). HRD cluster 1 harbored the highest tumor mutational burden as it had the highest aneuploidy scores and SNV neoantigen counts (Fig. 5H, I). Meanwhile, cluster 1 was genomically unstable since we found that fractions of genomic alteration and MSI Mantis scores were uniquely highest in cluster 1 (Fig. 5H, I). Regarding immune infiltration (signature), HRD cluster 1 showed the highest infiltrating abundance of M2 macrophage but the lowest cytotoxic T cells (Fig. 5H). And it showed the highest Th1/Th2 ratio bias to the adaptive immune infiltrate (Fig. 5H). Additionally, HRD cluster1 had the highest proliferation rate (Fig. 5H, I). The clonal evenness of TCR and BCR, the count of indel neoantigens, and CTA scores were not significantly different among the three HRD clusters (Fig. 5I).

### Regulation of immunomodulators

Immunomodulators (IMs) are critical for cancer immunotherapy, with numerous IM agonists and antagonists being evaluated in clinical oncology. To advance this research, understanding their expression and modes of control in different states of the tumor microenvironment (TME) is needed. We examined IM gene expression, CNVs, and SNVs.

Gene expression of IMs (Additional file 10: Table S7, Additional file 2: Fig. S2A) varied across HRD clusters, perhaps indicating their role in shaping the TME. Generally, most genes encoding IMs were at low expression levels in HRD cluster 1. Genes with the most significant differences between clusters (Additional file 2: Fig. S2B) included CX3CL1 (BH-adjusted p < 10^–5^), most lowly expressed in cluster 1 and TNFSF4 (BH-adjusted p = 0.004), most highly expressed in cluster 1. 

Copy-number variations affected multiple IMs and varied across HRD clusters. Cluster 1 showed both frequent amplification and deletion of IM genes, consistent with their greater genomic instability. In particular, BTLA was most frequently amplified in cluster 1, while TIGIT deletion was enriched in cluster 3 (Additional file 2: Fig. S2C). Overall, these marked differences in IM copy number may reflect more direct modulation of the TME by cancer cells. The observed differences in regulation of IMs might have implications for therapeutic development and combination immune therapies, and the multiple mechanisms at play in evoking them further highlight their biological importance.

### Dimensionality reduction and construction of the HRD signature

**Fig. 6:**
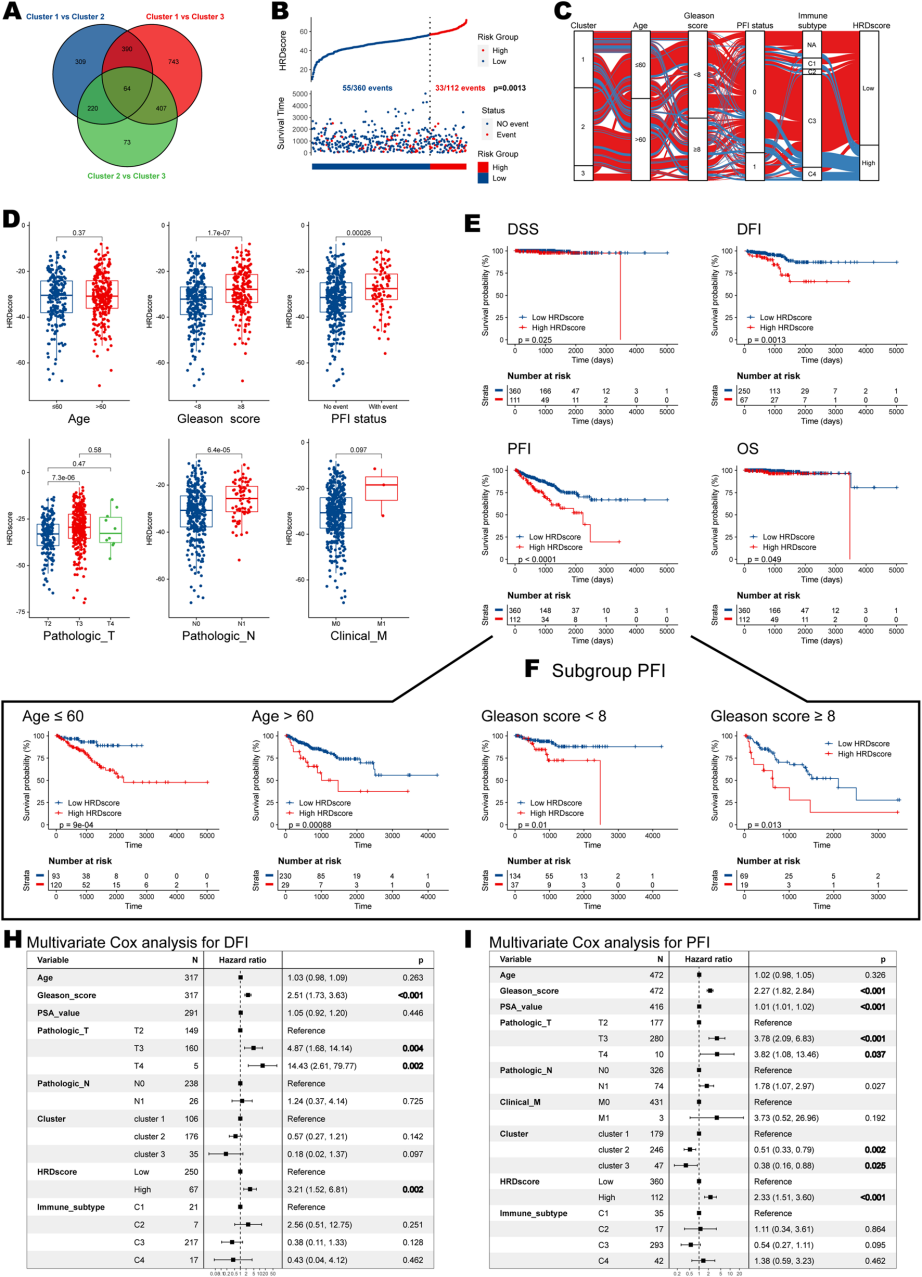
Construction and validation of HRDscore system. **A** Differentially expressed genes between pairs in HRD clusters. **B** Risk plot. The red dot meant sample with high risk, and the blue dot meant sample with low risk. **C** Sankey plot shows patients’ distribution in HRD clusters and HRDscore risk groups. **D** The HRDscore in different subgroups was stratified by age, Gleason score, PFI status, pathologic T and N stage, as well as clinical M stage. **E** The survival differences regarding DSS, DFI, PFI, and OS between high HRDscore and low HRDscore groups. **F** The survival differences regarding PFI between high HRDscore and low HRDscore groups in subgroups stratified by age (left) and Gleason score (right). **H**, **I** The results of multivariate Cox analyses for **H** DFI and **I** PFI. P value was set in bold if it was less than 0.05

To accurately quantify the prediction power of the HRD clustering, we applied a methodology to establish an HRD signature and calculate HRDscore for all the patients with prostate cancers. We obtained 64 prognostic hub genes when intersecting three group-paired differential expression analyses (DEGs) (Fig. 6A; Additional file 11: Table S8). Using the formula introduced in the “Method” section, we calculate the HRDscore for each patient based on the transcriptomic data. And we found that patients with higher HRDscores (threshold determined by X-tile) had a higher tendency of PFI event (recurrence, metastasis, or death) (55/360 vs. 33/112; p = 0.0013) (Fig. 6B). A Sankey plot was established to understand better the intrinsic relationship between HRD cluster and HRD signature (HRDscore) and other features for individuals (Fig. 6C). It intuitively showed that patients with higher HRDscores mostly came from HRD cluster 1, and they were predominantly classified with immune subtypes C3 (Inflammatory) and C4 (lymphocyte depleted). Subsequent specific analyses found that patients with higher Gleason scores (≥ 8), pathologic T and N stages, and those with PFI events had higher HRDscores (Fig. 6D), suggesting the ability of our HRD signature to represent malignant features.

An important finding of our study was that the established HRDscore could distinguish the survival outcome of patients with prostate cancers. Patients with higher HRDscore had worse prognosis when compared to those with lower scores, in the context of overall survival (OS) (p = 0.049), disease free survival (DSS) (p = 0.025), disease free interval (DFI) (p = 0.0013), and PFI (p < 0.0001) (Fig. 6E). Subgroup analyses stratified by patient’s age and Gleason score confirmed the stability of our HRD signature, as higher HRDscores consistently correlated with unfavorable prognosis (Fig. 6F). Furthermore, multivariate Cox analyses revealed the robustness of our signature since it showed that Gleason score, pathologic T stage, and HRDscore were independent predictors of patient’s DFI and PFI (Fig. 6H, I).

### HRDscore correlates with immunotherapy response, genomic instability, and immune infiltration

**Fig. 7:**
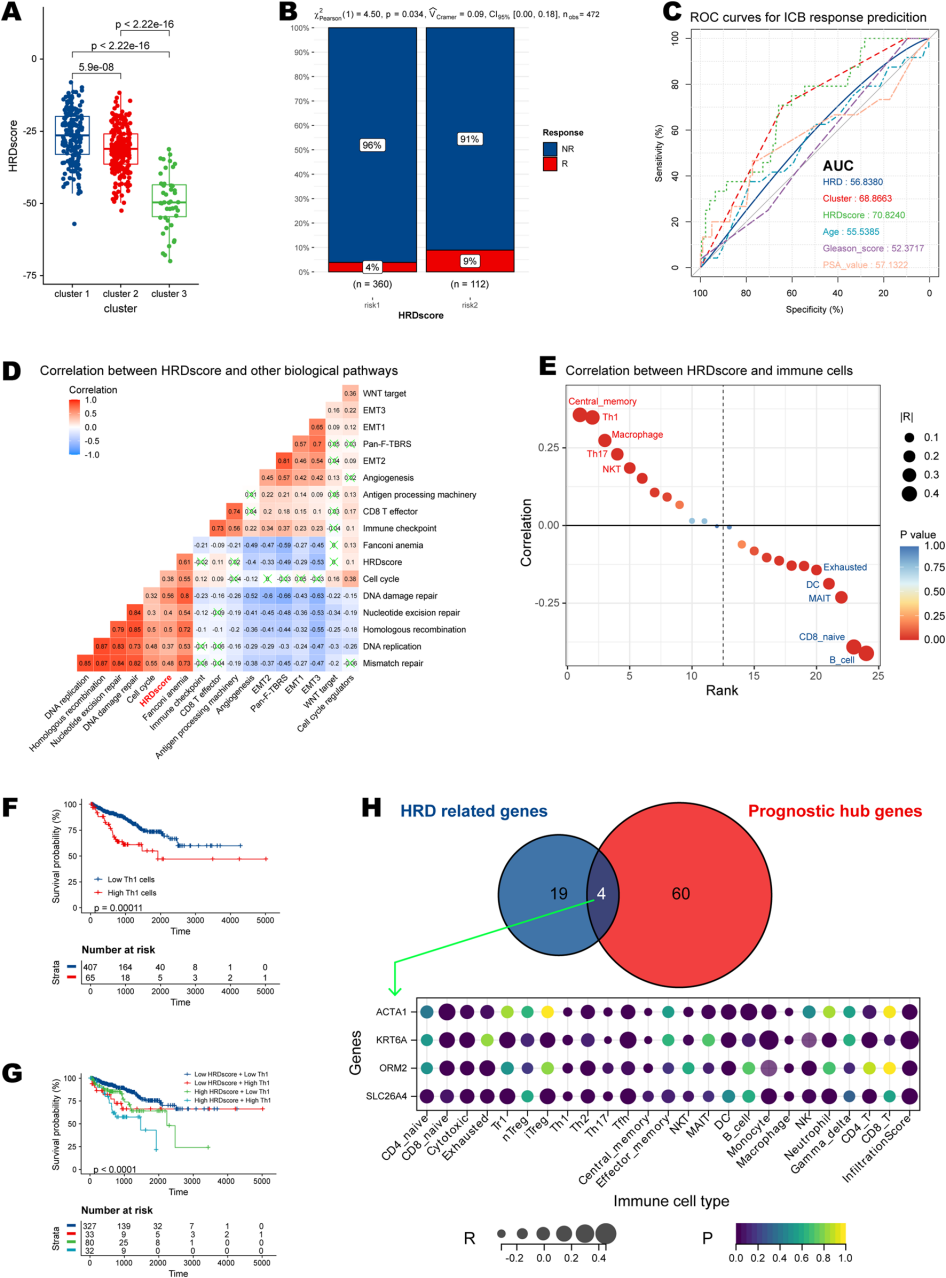
Correlation between HRDscore and immune signatures. **A** HRDscore in HRD clusters. **B** Response to immune checkpoint blockade in high and low risks. **C** ROC plot showing the power of response prediction of HRD to immune checkpoint blockade in high and low risks. **D** Correlation between HRDscore and existing famous biological pathways based on proteomic data. **E** Correlation between HRDscore and immune cells. **F** Kaplan–Meier survival plot of Th1 cell for PCa patients’ PFI. **G** Kaplan–Meier survival plot integrating Th1 cell and HRDscore for PCa patients’ PFI. **H** The four genes result from intersecting HRD related genes with prognostic hub genes and the correlation of their expression with immune infiltration

Patients previously assigned to HRD cluster 1 had the highest level of HRDscore (Fig. 7A). Using the bulk transcriptomic data, we acquired patient responses to ICB by ImmuneCellAI algorithm (Additional file 12: Table S9). High-HRDscore group demonstrated higher response rate to ICB than the low-HRDscore group (9% vs 4%, p = 0.034) (Fig. 7B). Besides, HRDscore outperformed other indicators in ICB response prediction with AUC = 70.82 (Fig. 7C). To better illustrate the characteristics of the HRD signature, we also tested the correlation between the known signatures and the HRDscore (Additional file 13: Table S10). After using the cluster method “ward.D2”, our HRD signature was clustered with recognized signatures related to genomic instability. In brief, it was strongly correlated with Fanconi anemia, cell cycle, DNA damage repair, Nucleotide excision repair, Homologous recombination, DNA replication, and mismatch repair (Fig. 7D).

Next, we explored the relationship between HRD signature and immune infiltration (Fig. 7E, Table). The HRDscore was positively correlated with central memory cells, Th1, Th17, macrophage, and natural killer T-cell (NKT). In contrast, it was negatively associated with B cell, CD8 naïve cell, dendritic cell (DC), and exhausted T cell. Univariate Cox analysis found that CD4 naïve T cell, exhausted T cell, and Th2 were protective cells, whereas Th1 was uniquely related to a poor prognosis in PCa patients (Additional file 14: Table S11). Specifically, patients with higher Th1 abundance had poor survival outcomes (Fig. 7F), and the effects were more significant when combined with the HRDscore group (Fig. 7G).

**Fig. 8:**
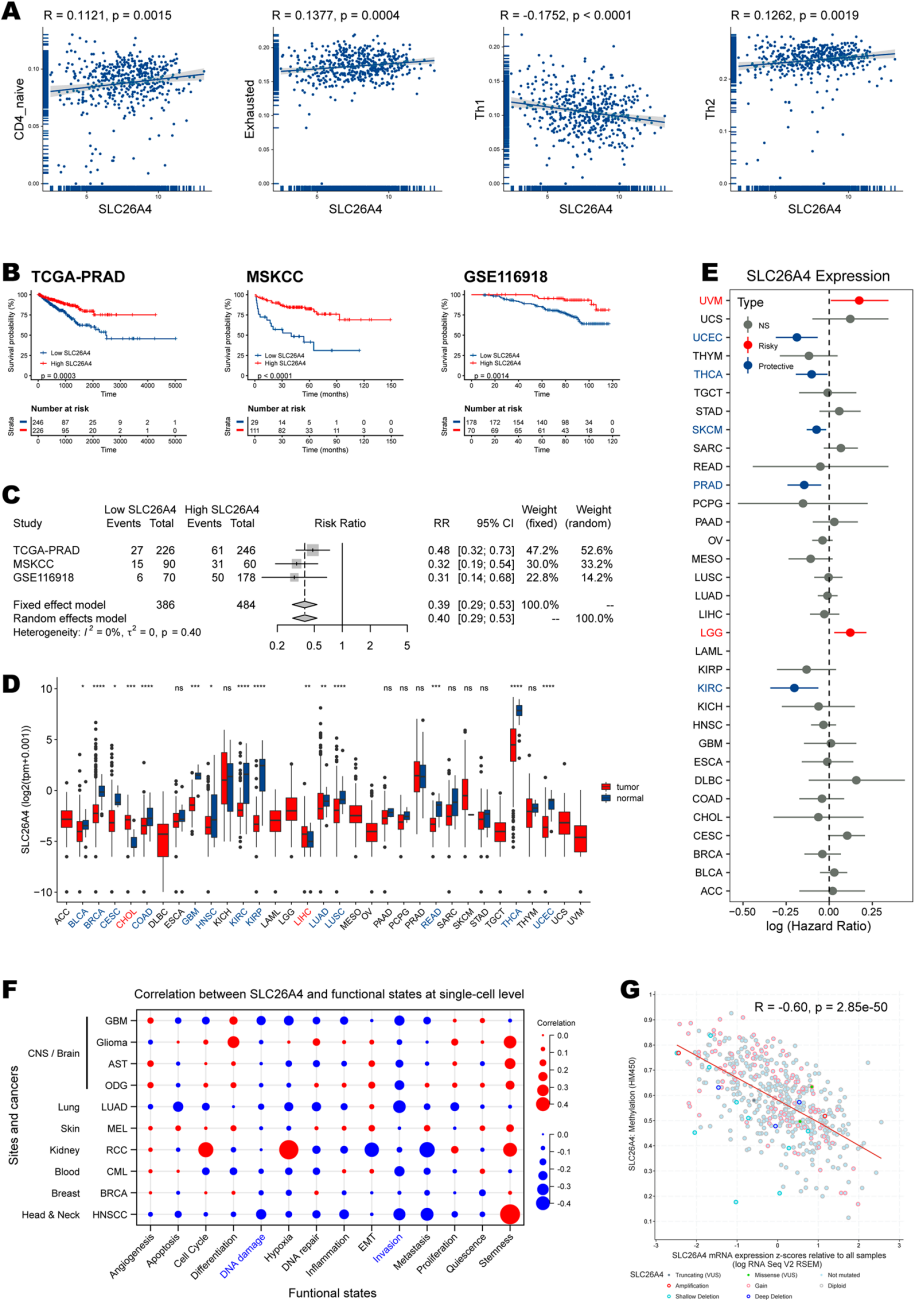
The results of multi-omics analysis of SLC26A4. **A** Correlations between SLC26A4 expression and abundances of immune cells, including CD4 naïve T cell, exhausted T cell, Th1, and Th2 cell. **B** Kaplan–Meier survival plots of SLC26A4 in TCGA-PRAD, MSKCC, and GSE116918 cohorts. **C** Forest plot of meta-analysis integrating SLC26A4’s role in these three datasets. **D** Expression levels of SLC26A4 between normal and tumor tissues in pan-cancer. **E** Correlations between SLC26A4 and survival in pan-cancer. **F** Correlations between SLC26A4 expression and functional states at the single-cell level. **G** Correlations between SLC26A4 expression and methylation. The statistical significance is indicated as asterisks (*), *p < 0.05, ** p < 0.01, ***p < 0.001, ns: not significant

To better focus on a single gene factor, we intersected HRD-related genes with prognostic hub genes, resulting in four candidate genes: actin alpha 1, skeletal muscle (*ACTA1*), keratin 6A (*KRT6A*), orosomucoid 2 (*ORM2*), and solute carrier family 26 member 4 (*SLC26A4*). We examined their links with 22 immune cells (Fig. 7H) and found that only SLC26A4 was significantly but not strongly correlated with the four prognostic immune cell types (CD4 naïve T cell, exhausted T cell, Th1, and Th2). The scatter plots illustrating correlations are displayed in Fig. 8A.

### SLC26A4 serves as a critical gene and correlates with immune infiltration and clinical prognosis

SLC26A4 was positively correlated with protective immune cells (CD4 naïve T cell, exhausted T cell, and Th2), while was negatively correlated with risk cell type (Th1) (Fig. 8A). Importantly, SLC26A4 consistently acted as a protective factor for PFI in TCGA-PRAD (p = 0.0003), MSKCC (p < 0.0001), and GSE116918 (p = 0.0014) cohorts (Fig. 8B). A meta-analysis revealed that, compared to low-SLC26A4 group, high-SLC26A4 group had a higher risk for PFI events (risk ratio = 0.39, 95% CI 0.29–0.53). And the pooled estimate showed no heterogeneity with *I*^*2*^ = 0% and tau-square = 0 (Fig. 8C).

Pan-cancer analysis focusing on SLC26A4 found that it was differentially expressed in most cancer types (Fig. 8D). Elevated in cholangiocarcinoma (CHOL) and liver hepatocellular carcinoma (LIHC) compared to corresponding normal tissues. Whereas SLC26A4 expression level was decreased in various tumor tissues in bladder urothelial carcinoma (BLCA), breast invasive carcinoma (BRCA), cervical squamous cell carcinoma and endocervical adenocarcinoma (CESC), colon adenocarcinoma (COAD), glioblastoma multiforme (GBM), head and neck squamous cell carcinoma (HNSC), kidney renal clear cell carcinoma (KIRC), kidney renal papillary cell carcinoma (KIRP), lung adenocarcinoma (LUAD), lung squamous cell carcinoma (LUSC), rectum adenocarcinoma (READ), thyroid carcinoma (THCA), and uterine corpus endometrial carcinoma (UCEC). Moreover, SLC26A4 was a risk for uveal melanoma (UVM) and LGG. It was protective for UCEC, THCA, SKCM, PRAD, and KIRC (Fig. 8E).

To better understand the function of this gene in cancer, we next obtained expression data and functional state scores at the single-cell level in the CancerSEA database (Additional file 15: Table S12). In all available cohorts, SLC26A4 has negatively correlated “DNA damage” and “invasion” functions (Fig. 8F). Unfortunately, the prostate cancer single-cell cohort there didn’t provide the expression data for SLC26A4, hampering our understanding of the correlations in prostate cancer. Finally, DNA methylation of SLC26A4 (by Illumina HumanMethylation450 BeadChip) was increased in prostate cancer tissues than the normal controls (Additional file 3: Fig. S3). The methylation was inversely correlated with its mRNA expression in TCGA-PRAD cohort (R = -0.60, p = 2.85e−50; Fig. 8G), suggesting epigenetic silencing. The methylation probe ID was cg15320854, and the methylation site was cpg 107660494.

### SLC26A4 was down-regulated in prostate cancer samples with HRD in independent external validation

Among the 62 included patients enrolled in Xiangya Hospital, nine (14.5%) were diagnosed with benign prostatic hyperplasia (BPH). Of the remaining 53 patients with prostate cancer, 33 carry HRD mutation (Additional file 16: Table S13).

**Fig. 9:**
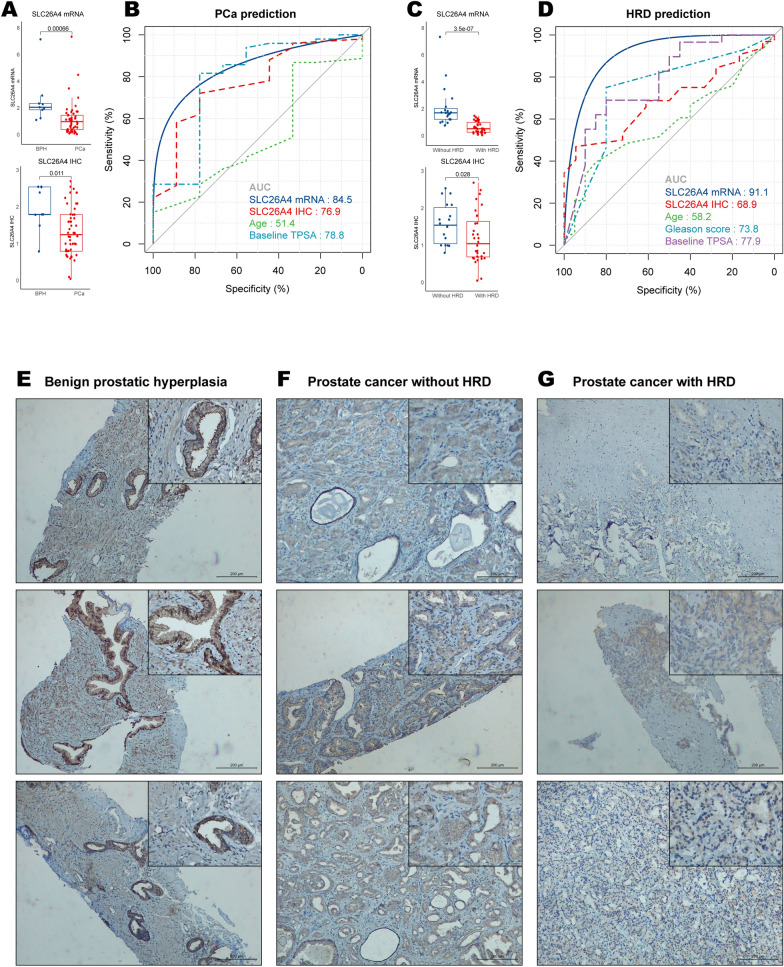
Validation of SLC26A4 in Xiangya cohort. **A** Expression difference of SLC26A4 mRNA and IHC between PCa and BPH tissues. **B** ROC curves of age, baseline PSA, and SLC26A4 mRNA and IHC in distinguishing PCa and BPH samples. **C** Expression difference of SLC26A4 mRNA and IHC between HRD and non-HRD PCa tissues. **D** ROC curves of age, Gleason score, baseline PSA, and SLC26A4 mRNA and IHC in distinguishing HRD and non-HRD PCa samples. Representative immunohistochemical results of **E** BPH, **F** HRD PCa, and **G** non-HRD PCa samples

Compared to BPH tissues, SLC26A4 mRNA expression levels and IHC scores were decreased in prostate cancer samples (Fig. 9A). SLC26A4 mRNA surpassed baseline total prostate specific antigen (PSA) value in predicting prostate cancer (AUC = 0.845; Fig. 9B). Furthermore, SLC26A4 was significantly down-regulated in prostate cancer tissues with HRD than those without HRD at mRNA and protein levels (Fig. 9C). And SLC26A4 demonstrated excellent performance in predicting HRD in the context of prostate cancer (AUC = 0.911; Fig. 9D). Representative IHC results in three tissue types are shown in Fig. 9E–G.

## Discussion

For many years, people have been exploring the initiation, development, and treatment of PCa. Gleason score and serum PSA level are still the most important prognostic factors of PCa. Recently, increasing evidence has suggested that HRD plays a key role in the biological process and therapeutic response in various tumors, one of the most influential factors for the prognosis of tumors [27]. In addition, many studies have found that mCRPC patients with HRD-related gene mutations show impressive responses to PARP inhibitors, even in very advanced disease settings [28–32]. The common HRD-related gene mutations in PCa are *BRCA2*, *ATM*, and *CHEK2*, all of which are included in the molecular eligibility criteria of virtually all PARP inhibitor trials involving mCRPC patients [33]. However, their germline mutations were found in 5–6%, 1–2% and 1–2% of mCRPC patients, respectively [8, 9].Therefore, new biomarkers need to be developed for molecular typing of PCa patients. In this study, we deeply analyzed the molecular characteristics of PCa patients with different HRD scores and identified a biomarker that could be complementary to the HRD scores.

HRD score integrates three indicators focusing on DNA-based genomic instability, which has been less explored in prostate cancer. The previous study has found that patients with primary prostate cancer have lower HRD scores, while patients with germline *BRCA2* mutations have higher HRD scores [34]. Since *BRCA2* mutation is the indication of two PARP inhibitors recently approved by the Food and Drug Administration (FDA) for the treatment of PCa, HRD score analysis may help improve treatment options. In this study, by analyzing the HRD scores of the TCGA-PRAD cohort, we obtained 23 genes associated with HRD scores, defined as HRD-related genes. We identified three molecular patterns with distinct clinicopathological characteristics based on these genes, and HRD cluster 1 was particularly correlated with worse clinicopathological types and poor prognosis.

The HRD clusters demonstrated distinct immune landscapes. In general, T cells, CD8 T cells, cytotoxic lymphocytes, and natural killer (NK) cells were less enriched in HRD cluster 1 indicating that the inhibited immune response may explain the poor outcome of patients in cluster 1. The HRD clusters also showed different immunogenomic characteristics. Specifically, HRD cluster 1 harbored the highest mutational burden, highest proliferation potentials, and lowest genomic stability, indicating an absolute potential to derive mutation and subsequent carcinogenesis. Besides, HRD cluster 1 demonstrated the lowest leukocyte abundance but the highest M2 macrophage infiltration. Macrophage infiltration in solid tumors is associated with poor prognosis [35]. The previous study has found that macrophages infiltrating PCa were mainly M2 type and associated with invasiveness and unfavorable outcome. We also noticed that Th1 to Th2 ratio was lowest in cluster 1. Cellular immunity mediated by Th1 mainly plays an anti-tumor role. Once it shifts from Th1 to Th2, resulting in immunosuppression. Thus, the anti-tumor immunity of the body will be seriously disturbed. Yamamura et al. and Kharkevitch et al. first found that Th2 cells were dominant in tumor patients [36, 37], and then found that Th2 shift occurred in many types of tumors such as non-small cell lung cancer, choriocarcinoma, glioma, gastric cancer, ovarian cancer, melanoma, colorectal cancer, and lymphoma. The above results define an immunosuppressive microenvironment phenotype of prostate cancer and an unstable genomic condition in HRD cluster 1.

Given the importance of IMs in cancer immunotherapy, we compared the differences in IM gene expression between these three clusters. The genes with the most obvious difference among clusters. In general, most of the IMs were in a relatively low expression level in HRD cluster 1 than those in clusters 2 and 3, suggesting that immune responses regulated by membrane checkpoints were less common there. Consistently, copy number variations of IMs were more frequent in HRD cluster 1 in amplification and deletion, confirming the unstable genomic phenotype. Although such a trend was not evident in SNV, several untypical checkpoints still had higher variation frequencies like *GZMA*, *PRF1*, *ENTPD1*, and *ARG1*. Paradoxically, TNFSF4 was significantly up-regulated in HRD cluster 1. Recent studies have shown that stimulation of OX40, the ligand of TNFSF4, is helpful for therapeutic immunization strategies for cancer [38]. It has been found that TNFSF4 is enriched in bone metastatic PCa [39]. Combined with our results, it may serve as a new therapeutic target in PCa, especially for those patients with high expression.

Furthermore, we established a signature (termed HRDscore) with excellent power to predict prognosis with stability. Based on proteomic data, the HRDscore was tightly correlated with existing signatures related to genomic instability, including homologous recombination, DNA damage repair, and Fanconi anemia (correlation coefficient ≥ 0.5, p < 0.001). This result suggested that the HRD-derived risk system could represent the signature of genomic defects. Besides, the HRDscore was positively related to macrophages, the unfavorable cell type, which was consistent with the above suppose. A recent article has explored HRD scores in PCa, which focused on the correlation between HRD scores and mutations of *BRCA2* and *ATM* [34]. However, these mutations are not common in PCa, especially in non-mCRPC, so the HRD score is of little value to numerous PCa patients without these mutations. In comparison, our HRDscore has excellent value for predicting the prognosis and even guiding treatment in PCa.

To further explore valuable biomarkers, we finally focused on a single gene, SLC26A4, correlated with immune infiltration and clinical diagnosis. It showed protective effects in several independent PRAD cohorts (RR 0.39, 95% CI 0.29–0.93, *I*^*2*^ = 0). Functional single-cell analysis suggested that SLC26A4 was negatively correlated with "DNA damage" and "invasion" functions. Nevertheless, the lack of prostate cancer single-cell cohort with SLC26A4 expression data hampered our understanding of its functions in PCa. Previous studies have mostly believed that SLC26A4 plays a vital role in maintaining normal hearing and never explored its significance in malignancies [40]. Our study revealed its potential value in tumorigenesis and development for the first time, which is worthy of in-depth exploration in future research.

SLC26A4 encodes a membrane protein called pendrin that permits the anion exchange between the cytosol and extracellular space, maintaining the proper function of auditory sensory cells. It is mainly expressed in the inner ear and thyroid gland, and its mutation is related to dyshormonogenic goiter and Pendred syndrome [41, 42]. Hypermethylation of SLC26A4 often occurs in cancers such as thyroid cancer and acute myoid leukemia [43, 44], consistent with our results. All the above findings indicated that the epigenetic changes of SLC26A4 may be involved in tumorigenesis. Our study uniquely found that SLC26A4 was highly associated with HRD in prostate cancer.

In our own Xiangya cohort, the SLC26A4 expression in PCa samples was lower than that in benign prostatic hyperplasia tissues at both mRNA and protein levels, which was inconsistent with the results of the TCGA-PRAD cohort. This may be due to the insufficient sample size of our cohort. Therefore, it needs to be further confirmed. Importantly, we found that SLC26A4 performed well in predicting HRD in patients with PCa. Patients with HRD-related gene mutations are often sensitive to PARP inhibitors, so we proposed that SLC26A4 may be a novel biomarker to screen patients sensitive to PARP inhibitors.

Consequently, we herein provided a potential biomarker for the treatment of PCa with PARP inhibitors. However, several limitations should be addressed in our study. First, there is a lack of SLC26A4 expression data in the prostate cancer single-cell cohort, which has been mentioned above. Secondly, our analyses were also limited by the relatively small sample size. Finally, due to the lack of prognostic and treatment information in the cohort, we failed to thoroughly verify the value of SLC26A4 in suggesting prognosis and guiding treatment. Therefore, further validation based on a large cohort is warranted.

## Conclusion

We introduced HRDscore to quantify the HRD pattern of individual PCa patients, which can predict the prognosis of PCa with stability and universality and has a specific value in guiding treatment. A new biomarker, SLC26A4, plays a protective role in PCa and can screen patients suitable for PARP inhibitor treatment.

## Supplementary Information

**Additional file 1: Figure S1.** Consensus clustering of HRD-related genes in the MSKCC-PRAD cohort. (Left) The consensus matrix when k = 3. (Right) The survival differences in three clusters regarding PFI.

**Additional file 2: Figure S2.** The immune checkpoint landscape in distinct HRD clusters. (A) Regulation of immunomodulators in distinct HRD clusters. From left to right: mRNA expression (median normalized expression levels); amplification frequency (the difference between the fraction of samples in which an immunomodulator is amplified in a particular subtype and the amplification fraction in all samples); and the deletion frequency (as amplifications) for 75 immunomodulatory genes by HRD clusters. (B) Representative mRNA expression levels (CX3CL1 and TNFSF4) in distinct HRD clusters. (C) Representative CNV frequency (of BTLA and TIGIT) in distinct HRD clusters. The statistical significance is indicated as asterisks (*), *p < 0.05, ** p < 0.01, ***p < 0.001, ns, not significant.

**Additional file 3: Figure S3.** The DNA methylation level of SLC26A4 in prostate cancer tissues and paired normal tissues in the TCGA-PRAD cohort.

**Additional file 4: Table S1.1.** HRD scores of PCa patients from the PANCAN cohort. **Table S1.2.** Differentially expressed genes.

**Additional file 5: Table S2.** Univariate Cox analysis results of differentially expressed genes.

**Additional file 6: Table S3.** The detailed CNV percentages of HRD-related genes in PCa.

**Additional file 7: Table S4.** Correlations among HRD-related genes.

**Additional file 8: Table S5.** Information of HRD clusters and patient survival.

**Additional file 9: Table S6.1.** ESTIMATE score. Table S6.2 Immune cell fractions based on ImmuCellAI algorithm. Table S6.3 Immune cell fractions based on ImmuCellAI algorithm. Table S6.4 Immune cell fractions based on CIBERSORT algorithm.

**Additional file 10: Table S7.** The mRNA expression of IMs in PCa.

**Additional file 11: Table S8.1.** Differentially expressed genes between HRD cluster 1 and cluster 2. **Table S8.2.** Differentially expressed genes between HRD cluster 1 and cluster 3. Table S8.3 Differentially expressed genes between HRD cluster 2 and cluster 3.

**Additional file 12: Table S9.** Estimated response to immune checkpoint blockades.

**Additional file 13: Table S10.** HRDscore and existing famous biological pathways based on proteomic data.

**Additional file 14: Table S11.** The correlation between immune cell type and patient survival based on Cox analyses.

**Additional file 15: Table S12.** Correlation between SLC26A4 expression and cancer functional states at single-cell level in multiple cancer types.

**Additional file 16: Table S13.** The clinical characteristics, pathological diagnosis, HRD gene mutation, and SLC26A4 expressions of the samples in Xiangya Cohort.

## Data Availability

The datasets used and/or analyzed during the current study are available from the corresponding author on reasonable request.
