# Supplementary figures and images for: SLC26A4 correlates with homologous recombination deficiency and patient prognosis in prostate cancer
Source: J Transl Med. 2022 Jul 14;20:313. doi: 10.1186/s12967-022-03513-5 (PMC9281181; doi:10.1186/s12967-022-03513-5)

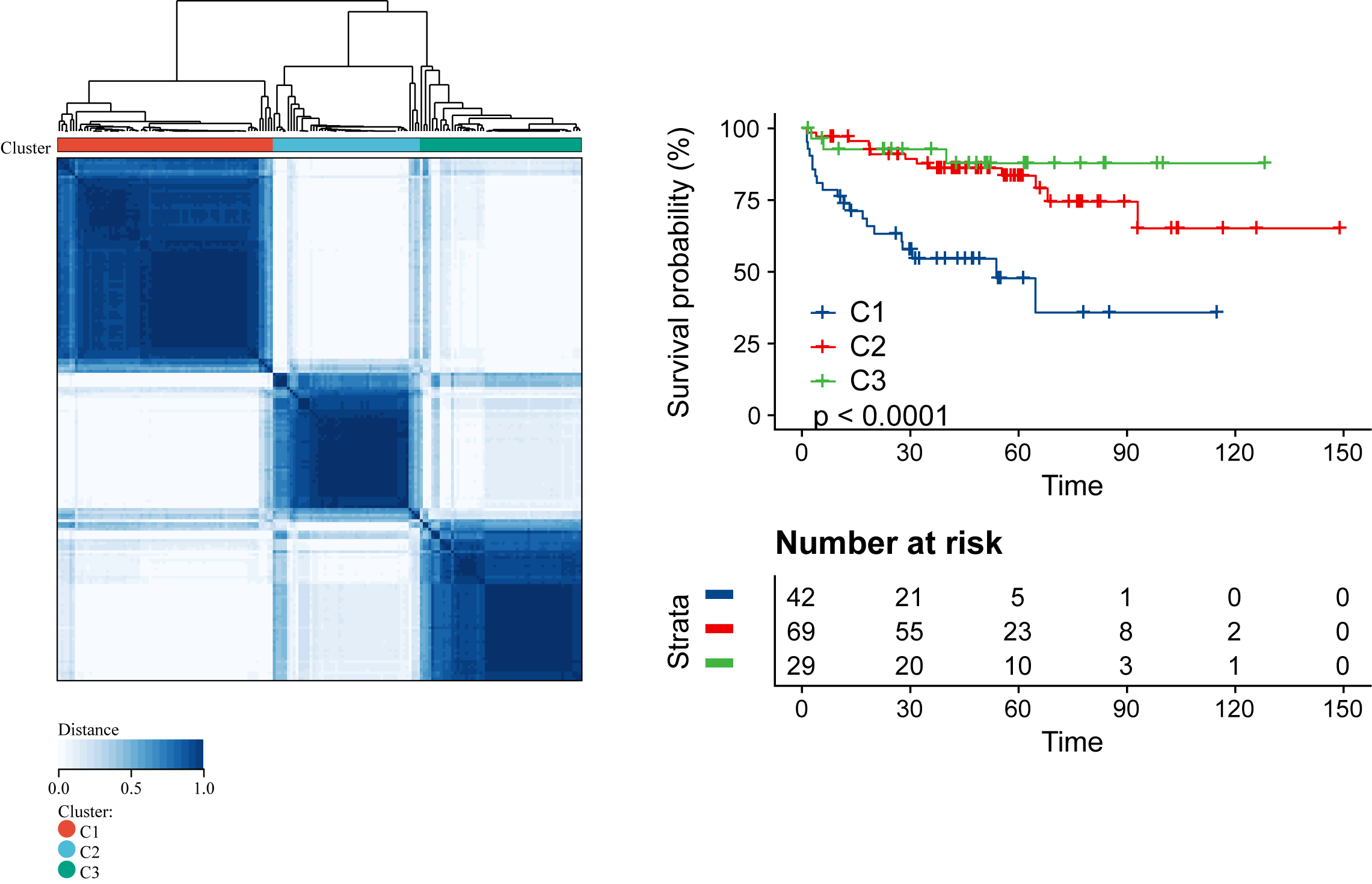

Supplement: Supplementary file 1 — Additional file 1: Figure S1. Consensus clustering of HRD-related genes in the MSKCC-PRAD cohort. (Left) The consensus matrix when k = 3. (Right) The survival differences in three clusters regarding PFI. [file 12967_2022_3513_MOESM1_ESM.tif]

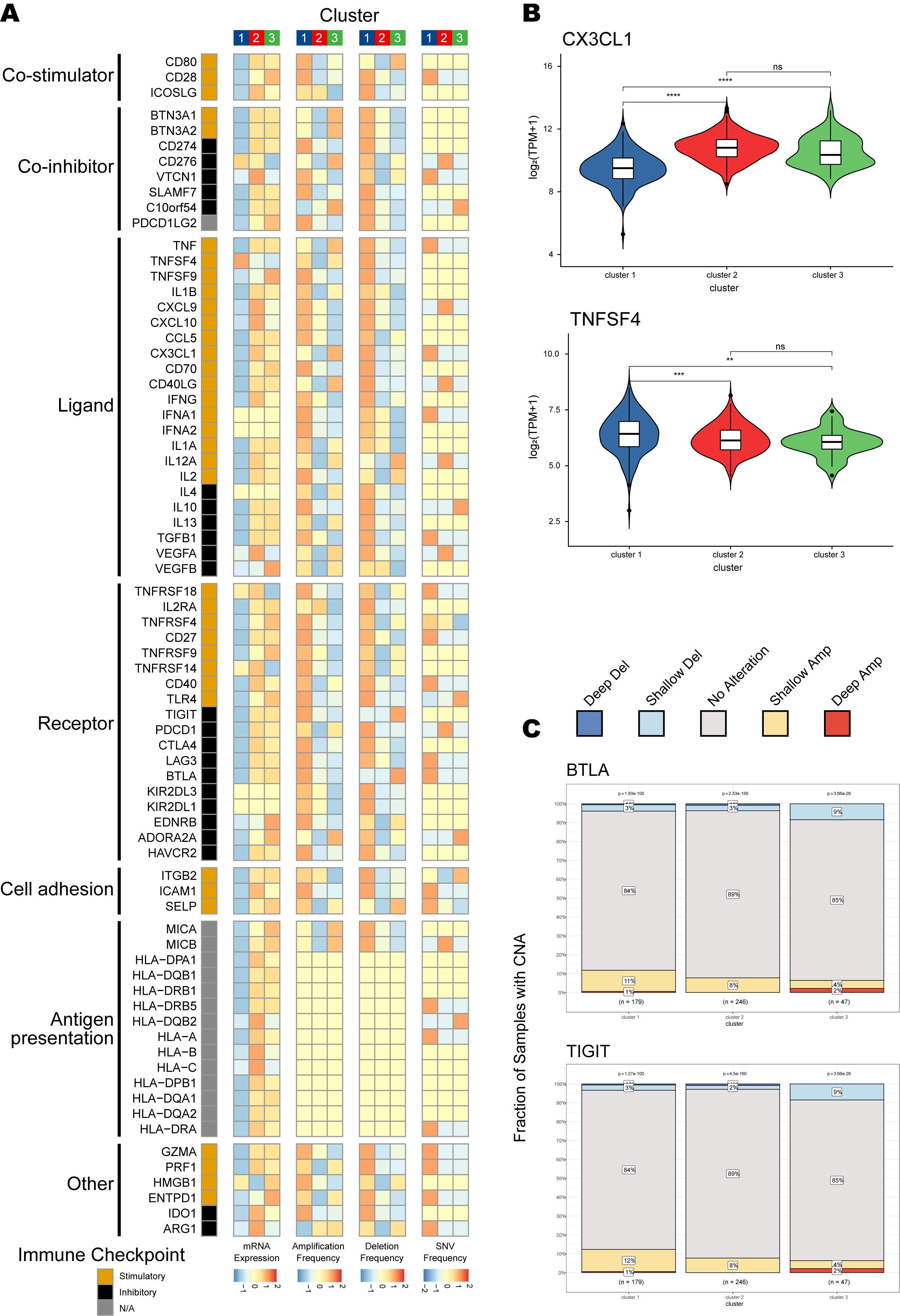

Supplement: Supplementary file 2 — Additional file 2: Figure S2. The immune checkpoint landscape in distinct HRD clusters. (A) Regulation of immunomodulators in distinct HRD clusters. From left to right: mRNA expression (median normalized expression levels); amplification frequency (the difference between the fraction of samples in which an immunomodulator is amplified in a particular subtype and the amplification fraction in all samples); and the deletion frequency (as amplifications) for 75 immunomodulatory genes by HRD clusters. (B) Representative mRNA expression levels (CX3CL1 and TNFSF4) in distinct HRD clusters. (C) Representative CNV frequency (of BTLA and TIGIT) in distinct HRD clusters. The statistical significance is indicated as asterisks (*), *p < 0.05, ** p < 0.01, ***p < 0.001, ns, not significant. [file 12967_2022_3513_MOESM2_ESM.tif]

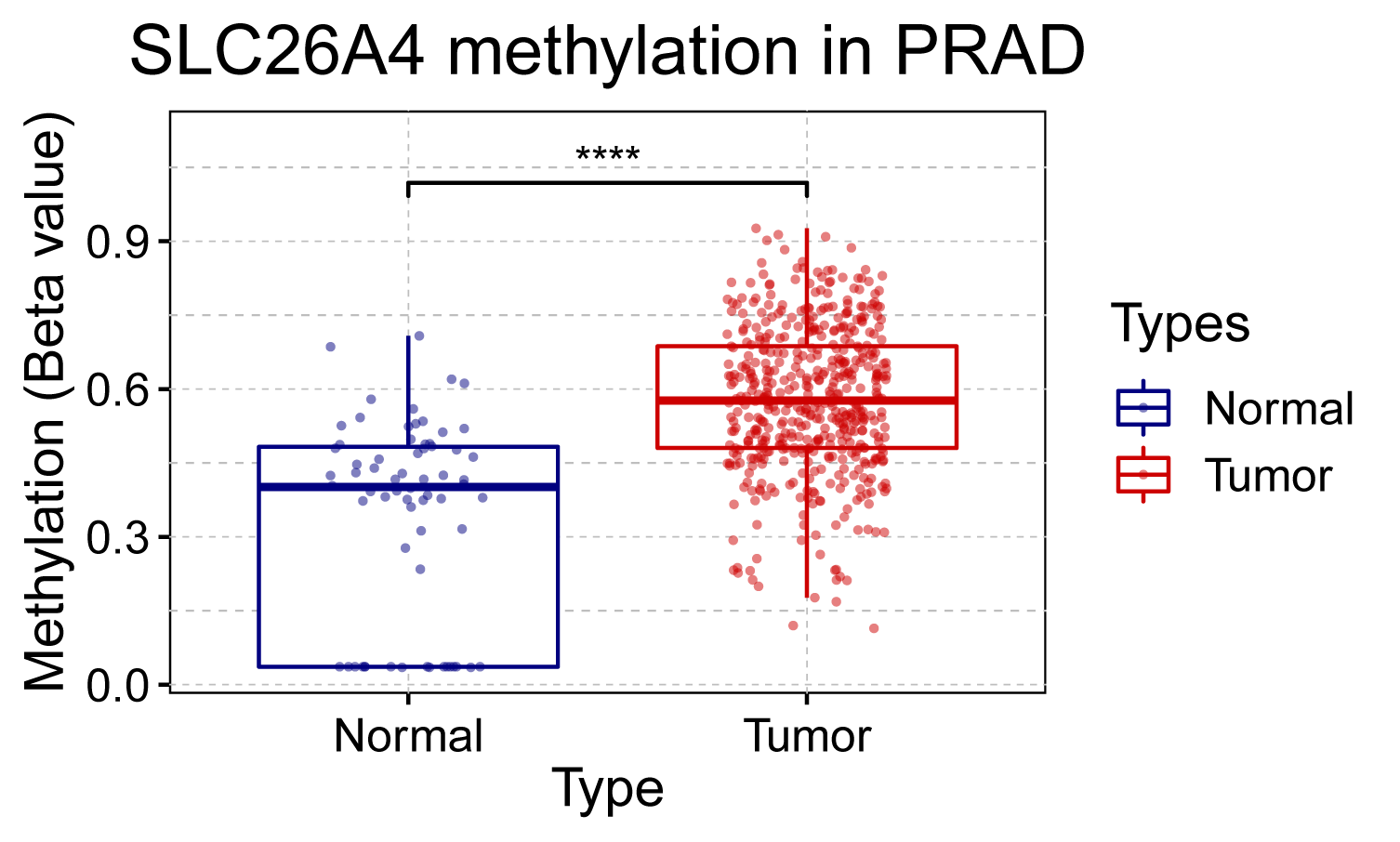

Supplement: Supplementary file 3 — Additional file 3: Figure S3. The DNA methylation level of SLC26A4 in prostate cancer tissues and paired normal tissues in the TCGA-PRAD cohort. [file 12967_2022_3513_MOESM3_ESM.tif]
